# Visually Perceived Negative Emotion Enhances Mismatch Negativity but Fails to Compensate for Age-Related Impairments

**DOI:** 10.3389/fnhum.2022.903797

**Published:** 2022-06-27

**Authors:** Jiali Chen, Xiaomin Huang, Xianglong Wang, Xuefei Zhang, Sishi Liu, Junqin Ma, Yuanqiu Huang, Anli Tang, Wen Wu

**Affiliations:** ^1^Department of Rehabilitation Medicine, Zhujiang Hospital, Southern Medical University, Guangzhou, China; ^2^Guangdong Province Work Injury Rehabilitation Hospital, Guangzhou, China

**Keywords:** negative emotion, mismatch negativity (MMN), ageing, automatic detection, fearful facial expression

## Abstract

**Objective**: Automatic detection of auditory stimuli, represented by the mismatch negativity (MMN), facilitates rapid processing of salient stimuli in the environment. The amplitude of MMN declines with ageing. However, whether automatic detection of auditory stimuli is affected by visually perceived negative emotions with normal ageing remains unclear. We aimed to evaluate how fearful facial expressions affect the MMN amplitude under ageing.

**Methods**: We used a modified oddball paradigm to analyze the amplitude of N100 (N1) and MMN in 22 young adults and 21 middle-aged adults.

**Results**: We found that the amplitude of N1 elicited by standard tones was smaller under fearful facial expressions than neutral facial expressions and was more negative for young adults than middle-aged adults. The MMN amplitude under fearful facial expressions was greater than neutral facial expressions, but the amplitude in middle-aged adults was smaller than in young adults.

**Conclusion**: Visually perceived negative emotion promotes the extraction of auditory features. Additionally, it enhances the effect of auditory change detection in middle-aged adults but fails to compensate for this decline with normal ageing.

**Significance**: The study may help to understand how visually perceived emotion affects the early stage of auditory information processing from an event process perspective.

## Introduction

The information detected by the auditory system far exceeds our brain’s attentive processing capacity in daily life. Thus, in the pre-attentive processing stage, the brain automatically monitors the environment outside the focus of conscious attention and can detect even small memory-based changes (Stefanics and Czigler, 2012; Stefanics et al., 2014), which allows for rapid and prioritized processing of salient stimuli. Mismatch negativity (MMN), one of the event-related potentials (ERPs) components, can be elicited by any discriminable change in a stream of auditory stimuli, reflecting a prediction error signal of an automatic change detection mechanism (Näätänen et al., 1978). It can be observed even in absence of attention, which has the advantage compared with other cognitive ERPs components such as P300 (P3), making it widely used in clinical patients (Sutton et al., 1965). MMN can be affected by many factors, among which the influence of visually perceived emotion is being studied more and more. However, the results from previous studies have not been consistent.

Emotion affects the perceptual processing of information and the allocation of attention resources (Lang et al., 1990, 1998). A recent review of emotion and attention demonstrated that attention to emotion (such as fear and anger) is associated with increased P3/LPP components in late information processing, but the emotional effects of ERP components in early information processing are inconsistent (Schindler and Bublatzky, 2020). Emotion perception involves two different attention mechanisms: top-down and bottom-up. Previous researches have shown that negative information has a more significant impact on cognition domains than positive information (Kisley et al., 2007). In particular, negative emotion helps in the detail-oriented analysis of information, such as fear, which tells people there may be potential threats in their environment and motivates them to take specific actions (Pourtois et al., 2004; Sugimoto et al., 2007). Up to now, various studies on the effect of visually perceived emotion on the automatic detection of auditory stimuli are not completely conclusive. Previous studies have observed that fearful images increased the MMN amplitude compared to neutral and positive images (De Pascalis et al., 2005). However, another study found the opposite result: the amplitude decreased when viewing negative photographs (Pinheiro et al., 2017). Besides, some studies found that MMN amplitude had no significant difference in different emotional contexts, indicating that MMN is not affected by emotional contexts (Lv et al., 2011). In a word, current studies have not reached a consistent conclusion on whether and how negative emotions affect the automatic processing of auditory stimuli.

For humans, facial expressions are the primary means of conveying and communicating emotions (Ohman et al., 2001). Studies of patients with visual impairment have shown that faces themselves are salient stimuli (Vuilleumier, 2000), especially faces with negative expressions such as anger and fear (Vuilleumier and Schwartz, 2001). In addition, the brain can spontaneously distinguish these emotions from neutral facial expressions (Pizzagalli et al., 2000). Therefore, we used emotional facial expression pictures from the Chinese Facial Affective Picture System to determine whether and how negative emotion modulates automatic detection of auditory stimuli.

The studies of auditory change detection have found another component involved with attention to auditory stimuli. N1 (N100) is a negative wave with a peak latency of around 100 ms after the auditory stimuli, presented with a frontocentral scalp distribution (Näätänen and Picton, 1987), which reflects the predictability of stimulus and can be affected by the level of attention. The amplitude of N1 was smaller on standard compared with high-deviant stimuli, consistent with the increased predictability of the stimulus (Seppänen et al., 2012; Kühnis et al., 2014). Previous studies using nonstartle tone probes have confirmed that the amplitude of N1 was affected by visually emotional pictures, with N1 amplitudes of high-deviant stimuli enhancing when viewing negative images compared to neutral and positive ones (Sugimoto et al., 2007). However, it has not reached a unanimous conclusion whether visually negative emotion promotes or destroys auditory feature extraction.

Previous studies have confirmed that age-related degeneration manifests in multiple aspects of cognitive functions. It is important to study the effect of ageing on early perceptual processing, which is the basis of higher-order cognitive processes (Cheng and Lin, 2012). MMN has been proposed as an index of the sensory memory trace, which provides a necessary premise for the automatic detection of auditory changes (Näätänen et al., 2007). In the field of the effect of ageing on MMN, previous studies have not been entirely conclusive. Studies using longer (>2 s) interstimulus intervals (ISIs) or stimulus onset asynchrony (SOA) found that the amplitude of MMN decreased in the elderly (Czigler et al., 1992; Cooper et al., 2006; Ruzzoli et al., 2012). It has been argued that the underlying mechanism is a faster decay of the sensory memory trace (Czigler et al., 1992; Cooper et al., 2006; Ruzzoli et al., 2012). In other words, the time that the elderly retain sensory memory representations decreases with age. However, Studies using short ISIs/SOA (<2 s) showed inconsistent results. Some studies found a smaller amplitude of MMN in the elderly (Alain and Woods, 1999; Cooper et al., 2006; Cheng et al., 2012), while some studies did not find the age-related decline (Pekkonen et al., 1993, 1996; Gunter et al., 1996; Ruzzoli et al., 2012). In addition, it is not clear whether the effect of negative facial expressions on automatic detection of auditory stimuli alters with normal ageing or even occurs in the early stage of ageing (45–59 years).

In conclusion, we aimed to understand how the task-relevant visually perceived negative emotions affect auditory change detection. We used oddball paradigms in young and middle-aged adults to obtain the ERPs when completing a visual task. Since other attributes of the stimuli in the two different emotional faces were consistent, the ERP difference of different emotional facial expressions can be interpreted as the moderating effect of emotion on auditory change detection ability. The MMN was the primary focus of our study. We hypothesize that the MMN amplitude decreases with ageing, similar to previous studies. However, the effect of negative facial expressions on the amplitude of MMN in the middle-aged adults is not known. We inferred that a high alert state caused by negative facial expressions would improve the attenuated auditory changes detection due to ageing. According to the hypothesis, the amplitude of MMN in middle-aged adults is smaller than that of young adults under neutral facial expressions. While under negative facial expressions, the amplitude of MMN in middle-aged adults will be larger than that of neutral facial expressions. The performance may be similar to that of young adults. We also analyzed the amplitude of N1 to standard and high-deviant tones to determine the effects of negative expression and ageing. We hypothesize that ageing impairs auditory feature extraction, so the N1 amplitude in middle-aged adults is smaller than that in young adults. Negative emotions may promote auditory processing, so N1 amplitudes are larger under negative facial expressions than neutral ones to standard tones.

## Materials and Methods

### Participants

We used G*Power (version 3.1) and selected the F test to calculate the prior sample size of MMN components at an alpha level of 0.05, the statistical power of 0.80 and a large effect size of 0.45. A total sample size of at least 36 was required. Forty-three healthy subjects recruited from the local university and community participated in the experiment, all of whom were right-handed. According to age criteria, they were assigned to two groups: the young adults (range: 20–44 years) and the middle-aged adults (range: 45–59 years). The final sample comprised 21 young adults (11 females, mean age = 26.0, SD = 2.96) and 20 middle-aged adults (11 females, mean age = 51.2, SD = 4.40). Participants who did not meet the following inclusion criteria were excluded: normal or corrected-to-normal vision, free of any hearing impairment, psychiatric or neurological disorders, more than 4 years of formal education, and a score equal or greater than 22 (the cutoff for mild cognitive impairment; Freitas et al., 2013) in the Montreal Cognitive Assessment (MoCA; Nasreddine et al., 2005). All participants gave written informed consent after they had been fully familiarized with the nature and procedure of the experiments. All of them received payment for their participation.

### Stimuli and Experimental Paradigm

**Figure 1:**
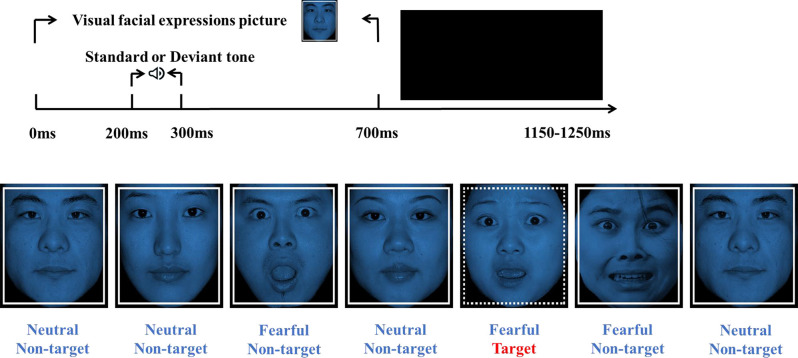
Experimental design and a visual stimuli example. The time course of the oddball paradigm (up). Each visual facial expression picture (Non-target/Target stimulation) was presented for 700 ms, followed by the ISIs between 450–550 ms randomly. At 200 ms after picture onset, a tone (Standard or High-deviant) was presented for 100 ms. Visual stimuli are composed of non-target stimulus (solid lines) and target stimulus (dotted lines; bottom). Neutral and fearful facial expressions were half of each type of stimuli.

Two tones were used in the oddball paradigm included 800 standard tones (1,000 Hz, 100 ms, 70 dB) and 200 high-deviant tones (2,000 Hz, 100 ms, 70 dB). Visual stimuli consisted of 10 fearful faces (5 males, 5 females) and 10 neutral faces (5 males, 5 females) pictures selected from the Chinese Facial Affective Picture System, evaluated by identity rate and intensity point (Gong et al., 2011). The index of identity rate refers to the percentage of the number of participants who think the picture belongs to this emotion type in the total number of participants. The intensity point corresponding to each picture refers to the average score of the emotional intensity assessed by all participants (1 is weakest, 9 is strongest). The values of the identity rate and intensity point of the pictures used in this study were as follows (mean ± standard deviation): neutral faces (identity rate: 82.50 ± 8.88, intensity point: 5.84 ± 0.17), fearful faces (identity rate: 77.33 ± 7.33, intensity point: 6.47 ± 0.86). The stimuli were presented in a pseudo-random manner, avoiding immediate repetition. All visual non-targets stimuli were framed with solid lines. The target stimuli were framed with dotted lines. There were 900 visual non-targets and 100 visual targets (neutral and fearful faces accounted for 50% of each type of stimulus; Figure 1).

Participants were seated on a comfortable chair in a sound-attenuated, dimly lit room. The oddball paradigm was designed and presented with E-prime 3.0 (Psychology Software Tools Inc., Pittsburgh, USA). Each picture (518*597 pixels) showed on the center of a 21-inch CRT monitor (60 Hz refresh rate) with a black background. The viewing distance was 100 cm. The vertical angle of each picture was 12.08°, and the horizontal angle was 10.48°. Each trial started with one facial expression (neutral or fearful), and at 200 ms after picture onset, a tone (standard or high-deviant) was presented for 100 ms. The picture duration was 700 ms, and the ISIs were randomized between 450 and 550 ms (Figure 1). Participants were asked to ignore the sounds and press the space button to detect the visual target stimulus quickly. The pictures and sounds are presented pseudo-randomly, avoiding immediate repetition. Only when the accuracy rate of the exercise block reached 100% indicated that participants had fully understood the study’s requirements would the formal block start. The formal experiment consisted of six blocks with 1,000 sounds and pictures. Each block lasted for about 4 min and a 1–2 min short break to allow participants to rest and adjust their posture.

### EEG Recording and Processing Procedures

Electroencephalogram (EEG) data were continuously recorded with 64 Ag/Ag-Cl scalp electrodes mounted according to the international 10–20 system and bilateral mastoids, using a BioSemi ActiveTwo system. The online band-pass was filtered by 0.16–100 Hz and sampled at 2,048 Hz. During recording, electrode impedance was maintained below 20 kΩ. EEG data were analyzed using an open-source EEGLAB toolbox (version 13_0_0b; Delorme and Makeig, 2004) in the Matlab (R2013b, MathWorks, Natick, MA, USA) development environment. We selected and located electrodes. An offline 50 Hz notch filter and 1–40 Hz off-line bandpass filter were applied to the EEG data. Previous MMN guidelines recommended that a 1 Hz high-pass filter can ideally reduce slow drift and high-frequency noise (Picton et al., 2000; Kujala et al., 2007; Duncan et al., 2009). The EEG signal was downsampled to 500 Hz and was segmented into epochs from 200 ms pre-stimulus to 1,000 ms post-stimulus and corrected to baseline (−200 to 0 ms). The epochs with obvious artifacts were removed and the Automatic Channel Rejection tool was used to identify bad channels and to interpolate these identified bad channels. Then we used independent component analysis (ICA) to inspect components that were related to eyeblinks or horizontal eye movements and remove them. Subsequently, epochs with potential values exceeding ±100 μV on any channel were excluded from data analysis. Each ERP was computed by averaging all trials for auditory stimuli in each condition. The grand average ERP waveform was obtained by merging and averaging all the data at the group level in each condition. For each stimulus of interest, the average of accepted trials was, for the auditory stimulation: 274.22 ± 28.28 (neutral standard), 298.25 ± 32.86 (fearful standard), 81.42 ± 7.75 (neutral high-deviant), and 57.61 ± 5.11 (fearful high-deviant).

### Statistical Analyses

#### Behavioral data

We used E-Prime 3.0 software to extract response time (RT) and accuracy of visual targets. After displaying visual stimuli, a correct button press within 100–700 ms was regarded as an accurate response. For RT, we only selected the trials that had the correct response. We performed a two-way repeated-measures analysis of variance (ANOVA) using SPSS 26 (IBM Corp, Armonk, NY, USA) for RT and accuracy, respectively, with emotion (neutral, fearful) as within-subject factor and group (young adults, middle-aged adults) as between-subject factor.

#### Event-Related Potentials

##### Auditory N100 (N1)

The N1 was the maximal negative deflection of about 100 ms after the presentation of each type of sound stimuli (i.e., about 300 ms after the appearance of the picture stimuli). Based on the previous studies (Näätänen and Picton, 1987; Sugimoto et al., 2007; Gulotta et al., 2013; Pinheiro et al., 2017), the grand-mean peak latency of the N1 was measured at the dominant site Fz (283 ms for neutral faces, 285 ms for fearful faces). The average amplitude of N1 was measured in a 40 ms time window centered on the grand-mean peak latency. The midline and lateral electrode sites were analyzed separately. For the midline site analysis, we performed four-factor ANOVAs with group (young adults, middle-aged adults), emotion (neutral, fearful), stimulus (standard, high-deviant), and electrode site (Fz, FCz, and Cz). For the lateral site analysis, the factor of the hemisphere was added, and ANOVAs with factors of group, emotion, stimulus, hemisphere (left, right), and site (F3/F4, FC3/FC4, C3/C4) were conducted. Subsequently, the effect of visually perceived emotion on each auditory stimulus type between two age groups was examined separately by two-way ANOVAs on the amplitude of N1 at the dominant site (Fz). The separate ANOVAs with emotion (neutral, fearful) as a within-subject factor and group (young adults, middle-aged adults) as a between-subject factor were conducted.

##### Auditory MMN

To examine the MMN, we selected auditory standard and high-deviant stimuli in the range of visual non-target stimuli trials for statistics, excluding data from visual target stimuli trials. Difference waves were obtained by subtracting the standard ERP waves from high-deviant ERP waves (i.e., neutral MMN = neutral high-deviant ERP waveform − neutral standard ERP waveform; fearful MMN = fearful high-deviant ERP waveform − fearful standard ERP waveform). Based on the previous studies (Duncan et al., 2009; Lv et al., 2011; Pinheiro et al., 2017), the MMN achieved the maximum negative deflection between 100 ms and 250 ms after the sound stimuli (i.e., 300–450 ms after the emotional facial expressions were displayed). The grand-mean peak latencies of neutral and fearful MMN were determined at each group’s electrode Fz. The mean amplitude of auditory standard and high-deviant stimuli were measured in a 40 ms latency window centered on the grand-mean peak latencies in each condition. Specifically, for young adults and middle-aged adults groups, the latency windows were 315–355 and 335–375 ms under neutral facial expressions and 315–355 and 305–345 ms under fearful facial expressions.

A four-factor ANOVAs analysis was performed with emotion (neutral, fearful), stimulus (standard, high-deviant), and site (F3, Fz, and F4) as within-subject factors, and group (young adults, middle-aged adults) as between-subject factor to determine whether MMN was successfully induced. Then, to explore whether the two facial expressions and age have different effects on MMN amplitude, we conducted three-factor ANOVAs analysis with emotion (neutral, fearful), and site (F3, Fz, and F4) as within-subject factors, and group (young adults, middle-aged adults) as between-subject factor. For each ANOVA, Bonferroni was used for *post hoc* comparisons. When necessary, the Greenhous-Geisser was used to correct the degree of freedom for non-sphericity. For significant results, the effect size was indicated by partial eta squared ($\eta_{p}^{2}$). The statistical significance was set at *p* = 0.050.

## Result

### Behavioral Results

For RT of visual target stimuli, we found a significant main effect of emotion (*F*_(1,34)_ = 5.920, *p* = 0.020, $\eta_{p}^{2}$ = 0.148). *Post hoc* comparisons showed that fearful facial expressions (442.67 ± 7.64 ms) had longer reaction times than neutral facial expressions (436.18 ± 6.95 ms). Neither the main effect of the group nor the interaction of emotion by group reached statistical significance.

**Figure 2:**
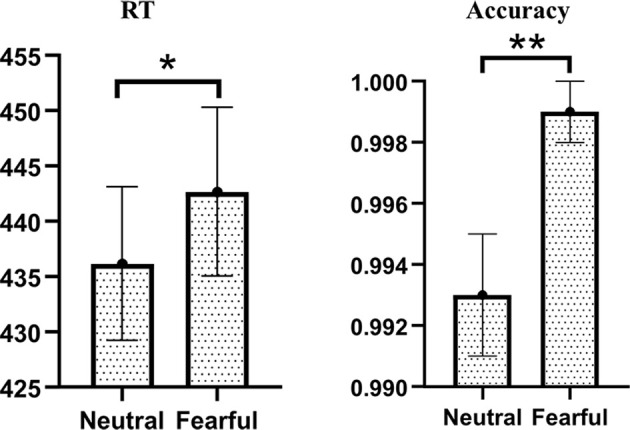
Behavioral results of visual target stimuli. There were significant main effects of Emotion on RT and accuracy. Compared with neutral facial expressions, the response of fearful facial expressions was slower but more correct (*p < 0.050; **p < 0.010).

The same analyses conducted for the accuracy also found the main effect of emotion was significant (*F*_(1,34)_ = 9.714, *p* = 0.004, $\eta_{p}^{2}$ = 0.222). *Post hoc* comparisons showed that fearful facial expressions (99.9% ± 0.1%) were more accurate than neutral facial expressions (99.3% ± 0.2%). Neither the main effect of the group nor the interaction of emotion by group reached statistical significance (Figure 2).

### Event-Related Potentials

#### Auditory N100 (N1)

The midline site analysis showed significant main effects of emotion, stimulus, site, and group (*F*_(1,34)_ = 4.473, *p* = 0.042, $\eta_{p}^{2}$ = 0.116; *F*_(1,34)_ = 14.906, *p* < 0.001, $\eta_{p}^{2}$ = 0.305; *F*_(2,68)_ = 23.100, *p* < 0.001, $\eta_{p}^{2}$ = 0.405; *F*_(1,34)_ = 4.701, *p* = 0.037, $\eta_{p}^{2}$ = 0.121, respectively). *Post-hoc* tests confirmed that the amplitude of N1 was significantly more negative at the Fz site (−7.436 ± 0.698 μV) and FCz site (−7.203 ± 0.631 μV) than at Cz site (−6.204 ± 0.504 μV), significantly larger for high-deviant stimuli (−7.218 ± 0.631 μV) than for standard stimuli (−6.678 ± 0.608 μV), and significantly larger in young adults (−8.262 ± 0.833 μV) than middle-aged adults (−5.633 ± 0.881 μV). The emotion × stimulus and stimulus × site interactions were also significant (*F*_(1,34)_ = 10.128, *p* = 0.003, $\eta_{p}^{2}$ = 0.230; *F*_(1,34)_ = 3.740, *p* = 0.029, $\eta_{p}^{2}$ = 0.099, respectively). For the lateral site analysis, a significant three-factor interaction between hemisphere, emotion and stimulus (*F*_(1,34)_ = 4.987, *p* = 0.032, $\eta_{p}^{2}$ = 0.128) indicated that the relation between hemisphere and emotion differed between standard and deviant stimuli. We thus calculated two-factor ANOVAs, including these variables separately for each type of stimulus, respectively. A significant interaction was obtained in the deviant stimuli (*F*_(1,35)_ = 5.399, *p* = 0.026, $\eta_{p}^{2}$ = 0.134). *Post hoc* tests confirmed that under fearful facial expressions, the N1 amplitude of the right hemisphere (−6.981 ± 0.656 μV) was more negative than that of the left hemisphere (−6.381 ± 0.633 μV, *p* = 0.003).

**Figure 3:**
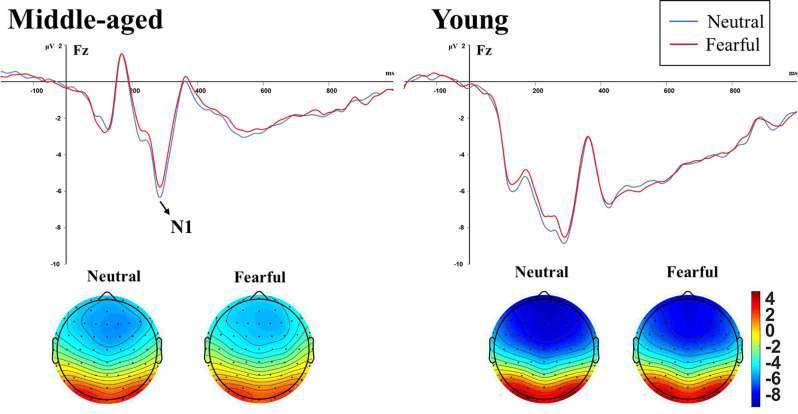
N1 waveform and topographic maps elicited by auditory standard stimuli under neutral and and fearful facial expressions in the middle-aged adults group (left) and young adults group (right). The amplitude was significantly modulated by Age and Emotion independently.

The subsequent separate ANOVAs for auditory standard and high-deviant stimuli at Fz site showed that the main effect of emotion and group on N1 amplitude was significant only for standard stimuli (*F*_(2,68)_ = 23.100, *p* < 0.001, $\eta_{p}^{2}$ = 0.405; *F*_(1,34)_ = 4.701, *p* = 0.037, $\eta_{p}^{2}$ = 0.121, respectively). The amplitude of N1 was significantly smaller for fearful facial expressions than for neutral facial expressions, and significantly larger in young adults than middle-aged adults. The amplitude of N1 for high-deviant stimuli was not affected by emotion and age (*F*_(1,34)_ = 0.033, *p* = 0.857, $\eta_{p}^{2}$ = 0.001; *F*_(1,34)_ = 4.061, *p* = 0.052, $\eta_{p}^{2}$ = 0.107, respectively). Table 1 showed the amplitude values and Figure 3 showed the waveforms of N1 components induced by standard stimuli.

**Table 1:** Mean amplitudes of N1 components (at Fz site) elicited by auditory standard stimuli for each emotion (μV with SD) in young and middle-aged adults.

Auditory standard	N1
**Middle-aged**	
Neutral	−5.94 (3.77)
Fearful	−5.43 (3.97)
**Young**	
Neutral	−8.74 (4.50)
Fearful	−8.33 (4.42)

#### Auditory MMN

Table 2 showed the amplitude values of auditory standard and high-deviant stimuli in each condition. For four-factor ANOVAs analysis, the emotion × stimulus interaction was significant (*F*_(1,34)_ = 10.113, *p* = 0.003, $\eta_{p}^{2}$ = 0.229). We examined the simple effects of emotion and stimulus separately. Under both neutral and fearful facial expressions, the amplitude of high-deviant stimuli was more negative than that of standard stimuli. The amplitude of difference waveform under fearful expression was larger than that under neutral expression (neutral: *p* < 0.001; fearful: *p* < 0.001). In both standard and high-deviant stimuli, the amplitude under the fearful facial expressions was more negative than the neutral facial expressions (standard: *p* = 0.001; high-deviant: *p* < 0.001). A significant group × emotion interaction was found (*F*_(1,34)_ = 26.713, *p* < 0.001, $\eta_{p}^{2}$ = 0.440). The simple effects analyses revealed a larger amplitude for fearful expression than neutral expression in middle-aged adults (*p* < 0.001). Under both neutral and fearful facial expressions, the amplitude of the young adults was more negative than that of the middle-aged adults (neutral: *p* < 0.001; fearful: *p* = 0.030). We also found that stimulus × group interaction was significant (*F*_(1,34)_ = 5.868, *p* = 0.021, $\eta_{p}^{2}$ = 0.147). The simple effect analysis showed that high-deviant stimuli elicited more negative amplitude in young and middle-aged adults than standard stimuli (young: *p* < 0.001; middle-aged: *p* < 0.001). The amplitude in young adults was larger than middle-aged adults in both standard and high-deviant stimuli. In addition, we also found that the main effect of site was significant (*F*_(2, 68)_ = 3.865, *p* = 0.026, $\eta_{p}^{2}$ = 0.102). The amplitude was more negative at the F3 site than at the Fz site (*p* = 0.012).

**Table 2:** Mean amplitudes of standard and high-deviant stimuli for each auditory condition (μV with SD) in young adults and middle-aged adults.

	Site	Standards	High-deviants
**Middle-aged**		
Neutral	F3	−0.97 (1.99)	−1.84 (2.13)
	Fz	−0.36 (2.02)	−1.09 (1.75)
	F4	−1.04 (2.34)	−1.67 (2.07)
Fearful	F3	−2.57 (2.62)	−3.97 (2.51)
	Fz	−2.34 (2.62)	−3.52 (2.79)
	F4	−2.73 (2.59)	−4.07 (2.92)
**Young**			
Neutral	F3	−5.28 (4.24)	−6.83 (4.61)
	Fz	−5.16 (4.25)	−6.57 (4.72)
	F4	−5.16 (4.06)	−6.61 (4.34)
Fearful	F3	−4.85 (4.12)	−7.04 (4.50)
	Fz	−4.81 (4.27)	−6.94 (4.65)
	F4	−4.58 (4.12)	−7.21 (4.52)

**Figure 4:**
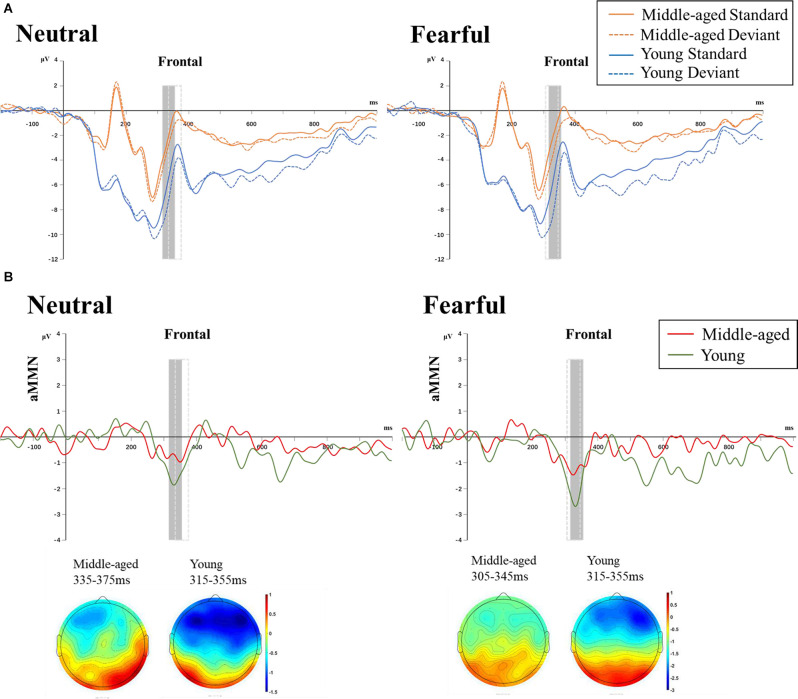
**(A)** ERPs induced by auditory standard and deviant stimuli under neutral (left) and fearful (right) facial expressions in the two age groups at frontal channels (averaged across F3, Fz, F4). **(B)** Difference wave (neutral MMN = neutral deviant − neutral standard; fearful MMN = fearful deviant − fearful standard) and topographic maps of the two age group under neutral (left) and fearful (right) expressions. The gray box indicated the time window for young adults and the dotted box for middle-aged adults. The main effect of Emotion and Age were significant. Note: All ERP waveforms were filtered by a 25 Hz low-pass filter only for graphical display.

The results of three-factor ANOVAs analysis of the MMN amplitude showed that the main effect of emotion and group were significant (*F*_(1,34)_ = 10.120, *p* = 0.003, $\eta_{p}^{2}$ = 0.229; *F*_(1,34)_ = 5.868, *p* = 0.021, $\eta_{p}^{2}$ = 0.147). The result of the *post hoc* comparison showed that the MMN amplitude of young adults (−1.894 ± 0.247 μV) was more negative than that of middle-aged adults (−1.024 ± 0.261 μV). The MMN amplitude of fearful facial expressions (−1.814 ± 0.210 μV) was more negative than that of neutral faces (−1.105 ± 0.213μV; Figure 4).

## Discussion

We used the modified oddball paradigm to explore the effects of visually perceived negative emotion on the automatic detection of auditory stimuli with early normal ageing. The ERP results confirmed our hypothesis that normal ageing attenuated automatic detection of auditory stimuli, while visually perceived negative emotion promoted automatic detection of auditory stimuli but was not enough to compensate for the reduction caused by ageing. We found that MMN amplitude under fearful facial expressions was larger than that of neutral facial expressions, but the amplitude in middle-aged adults was smaller than in young adults. In addition, ageing and visually fearful expressions significantly affected the N1 amplitude induced by standard stimuli. These results suggest that normal ageing and visually perceived emotional valence affect feature extraction and automatic detection of auditory input.

Some studies have confirmed that fearful expressions have a generally low identity rate (Wang and Markham, 1999; Wang and Luo, 2005). The previous studies have researched the development of expression judgment ability and found that the identity rate when recognizing medium and low intensity fear expression reached 40%, which was already regarded as a representative picture (Qiao, 1998; Gong et al., 2011). Our study found that the amplitude of N1 was smaller under fearful facial expressions than that of neutral, and the amplitude of MMN was more negative under fearful facial expressions than that of neutral ones. The two kinds of auditory input stimuli under different emotions have the same characteristics except for the different emotional categories, which confirms that the ERP components were affected by visually perceived emotion.

There are two essential processes in the formation of MMN. The first is the detection of the regularity of auditory information flow, and the second is the detection when the sensory input does not match the expectation (Pinheiro et al., 2017). Studies using the passive oddball paradigm to analyze the auditory standard and deviant stimuli responses respectively have found that the N1 component reflects the acoustic coding of stimuli (Näätänen and Picton, 1987) and is sensitive to the degree of selective attention (Woldorff and Hillyard, 1991; Tartar et al., 2012). The effect of predictability on N1 amplitude was reflected in the reduction of N1 amplitude induced by standard stimulus, which corresponded to the increase of sensory predictability of stimulus (Bendixen et al., 2012; Knolle et al., 2013; Timm et al., 2016). Similarly, some studies have found that N1 amplitude induced by standard stimulus decreases compared with the high-deviant stimulus. In line with previous studies, we found that the amplitude of N1 was significantly smaller for auditory standard stimuli than high-deviant stimuli (Seppänen et al., 2012; Kühnis et al., 2014). Our results showed that the N1 elicited by standard tones was significantly smaller (less negative) under fearful facial expressions than neutral facial expressions. The decrease in N1 amplitude induced by standard stimuli corresponded to the increase in sensory predictability, so our results supported that fearful facial expressions promoted the extraction of auditory features. This is different from previous studies, including one that showed the N1 amplitude induced by standard stimuli were larger in negative images than in neutral ones (Tartar et al., 2012). However, other studies have shown that N1 amplitude was unaffected by emotion (Pinheiro et al., 2017).

To sum up, the mechanism of the effect of emotion on N1 amplitude is far from clear, and more research is needed to support the current conclusions. A negative mood is generally influenced by low serotonin function (Mitchell and Phillips, 2007). Previous studies have demonstrated that the primary auditory cortex receives intensive serotonin projections that inhibit neural activity in this region (Hegerl and Juckel, 1993; Hegerl et al., 2001) and seem to be associated with lower N1 amplitude (Sugimoto et al., 2007). In addition, the amplitude of N1 was significantly more negative for young adults than middle-aged adults, which confirmed that normal early ageing affected N1 amplitude. A study comparing the electrical brain performance of musicians and non-musicians during auditory tasks found that musicians showed enhanced early N1, which was significantly associated with the years of music practice. It can be explained by the neuroplasticity process produced by musical training, with musicians being more efficient at anticipating auditory sensations (Bianco et al., 2021).

We successfully induced difference waves under neutral and fearful facial expressions, as the amplitude induced by the high-deviant stimuli was always more negative than the standard stimuli. The MMN component reflects the brain’s automatic processing, which is generated when the input tone’s frequency, duration, or intensity does not match the expected signal. The amplitude increases as the mismatch increases (Näätänen, 2000). Our result showed that fearful facial expressions modulated the amplitude of MMN. Under instructions to ignore the sound, the MMN amplitude was more negative under the fearful faces than the neutral faces. Our findings differed from previous studies that used intensity and duration as features and found that the amplitude of MMN decreased in the negative block relative to the neutral and positive block and was associated with self-reported mood (Pinheiro et al., 2017). The cognitive psychology theory confirms that positive emotional states expand the scope of attention while negative emotional states limit the scope of attention (Fredrickson, 2001). A negative state may mean a threatening environment that needs highly focused attention to act in time to escape from danger. In our experiment, fearful facial expressions induced larger MMN amplitude, possibly because fearful facial expressions made subjects more alert and easier to detect when the auditory input signal did not match the expected signal from the brain. More rigorous experimental design, such as comparing different auditory features or different visual stimulus materials (emotional contexts or emotional face images), is needed in the future to verify the conclusions. In summary, these findings suggested that the emotional valence of the visual stimuli influenced early perceptual processing.

We observed a larger negative amplitude in the middle-aged adults under fearful faces than under neutral faces. Importantly, the MMN amplitude in middle-aged adults was smaller than in young adults under both fearful and neutral facial expressions. Recently, a meta-analysis study (Cheng et al., 2013) has confirmed that the amplitude of MMN is also reduced in the elderly at short ISIs/SOAs. Our result using a short ISI/SOA that the MMN amplitude for frequency change showed the effect of age also supported this conclusion, suggesting that the decrease of MMN amplitude already occurred in the early stage of ageing. These results were similar to Czigler et al. (1992) results with ISIs of 800, 2,400 or 7,200 ms and to Woods’s (1992) results with SOAs of 200 and 400 ms. However, it contrasted with some studies that did not find the effect of age (Pekkonen et al., 1993, 1996; Gunter et al., 1996). A contributing factor is likely to be an age-related change in a mismatch processing (Alain and Woods, 1999). Middle-aged adults, for example, were less sensitive than younger adults in distinguishing between the frequency of standard and deviant stimuli. According to previous research on hearing, higher-frequency tones lose more rapidly and more severely with age than lower-frequency tones (Hickish, 1989). To be specific, in the middle-aged people aged 50–59, the first time that the faster loss is at 2,000 Hz. Besides, Pekkonen et al. (1993) and Pekkonen et al. (1996) adjusted stimulus loudness according to subjects’ hearing threshold, and MMN results found no age effect, while in the present study and Czigler et al.’s (1992) study, both fixed stimulus loudness was used and age effect was found. Pekkonen et al. (1996) argued that the decline in the inner ear’s ability to convert sound signals into electrical ones with age, reflected in weaker ERP responses in the cerebral cortex, might partly explain the differences in the experimental results. To sum up, we found that visually perceived negative emotion enhanced the automatic detection of auditory stimuli but did not alleviate the amplitude reduction associated with ageing.

### Study Limitations

There are several limitations to this study. We did not include positive facial expressions materials in the study. Neutral and fearful facial expressions accounted for half of each stimulus type, and the duration increased if positive facial expressions were added. Considering the simplicity of the task, if the duration of the experiment is increased, fatigue may be introduced to middle-aged adults. In addition, we may apply this paradigm to patients with neurological impairment. In order to ensure the comparability of the paradigm, we finally selected the negative emotional materials with more significant effects on attentional function (Kisley et al., 2007). Since the age coverage of the subjects is not broad enough, our conclusions should be treated with caution, and we are considering the inclusion of older subjects. Previous studies (Bianchin and Angrilli, 2012; Maffei et al., 2015, 2019) have confirmed significant individual differences in empathy ability, which affected the participants’ emotional response processing. Besides, there are substantial differences in empathy between males and females. Future studies need to assess subjects’ empathy traits to control for potential confusion.

## Conclusion

Visually perceived negative emotion promotes the extraction of auditory features, and it enhances the effect of auditory change detection in middle-aged adults but fails to compensate for this decline with normal ageing.

## Data Availability Statement

The original contributions presented in the study are included in the article, further inquiries can be directed to the corresponding author.

## Ethics Statement

The studies involving human participants were reviewed and approved by the Ethics Committee of Zhujiang Hospital Affiliated to Southern Medical University. The patients/participants provided their written informed consent to participate in this study. Written informed consent was obtained from the individual(s) for the publication of any identifiable images or data included in this article.

## Author Contributions

JC, WW, and XW contributed to conception and design of the study. JC and XH gathered experimental data. JC and SL performed the statistical analysis. JC wrote the first draft of the manuscript. WW, JM, and YH supervised and directed the research projects. JC, AT, and XZ contributed to manuscript revision. All authors contributed to the article and approved the submitted version.

## Conflict of Interest

The authors declare that the research was conducted in the absence of any commercial or financial relationships that could be construed as a potential conflict of interest.

## Publisher’s Note

All claims expressed in this article are solely those of the authors and do not necessarily represent those of their affiliated organizations, or those of the publisher, the editors and the reviewers. Any product that may be evaluated in this article, or claim that may be made by its manufacturer, is not guaranteed or endorsed by the publisher.
